# Odontogenic tumours in Nigeria: A multicentre study of 582 cases and review of the literature

**DOI:** 10.4317/medoral.22473

**Published:** 2018-11-21

**Authors:** Babatunde Aregbesola, Olujide Soyele, Olajumoke Effiom, Olalekan Gbotolorun, Olanrewaju Taiwo, Ibiyinka Amole

**Affiliations:** 1 Senior Lecturer/Consultant, Department of Oral and Maxillofacial Surgery and Oral Pathology, Faculty of Dentistry, Obafemi Awolowo University, Ile-Ife, Nigeria; 2 Lecturer/Consultant, Department of Oral and Maxillofacial Surgery and Oral Pathology, Faculty of Dentistry, Obafemi Awolowo University, Ile-Ife, Nigeria; 3 Senior Lecturer/Consultant, Department of Oral and Maxillofacial Pathology/Biology, College of Medicine, University of Lagos, Lagos, Nigeria; 4 Associate Professor/Consultant, Department of Oral and Maxillofacial Surgery, College of Medicine, University of Lagos, Lagos, Nigeria; 5 Senior Lecturer/Consultant, Department of Surgery, College of Health Sciences, Usmanu Danfodiyo University, Sokoto, Nigeria; 6 Senior Lecturer/Consultant, Department of Surgery, College of Health Sciences, Usmanu Danfodiyo University, Sokoto, Nigeria 6 Senior Lecturer/Consultant, Department of Oral and Maxillofacial Surgery, Faculty of Dentistry, Bayero University, Kano Nigeria

## Abstract

**Background:**

The objective of this study was to classify the various types of odontogenic tumours (OTs) using the newly updated 2017 world health organization (WHO) histological typing and to analyze the prevalence of these tumours among Nigerians as well as to compare the results obtained with reports from world-wide studies.

**Material and Methods:**

The records of four major tertiary hospitals in Nigeria were reviewed over a 12-year (2004-2015) period. Lesions diagnosed as odontogenic tumours were classified into four groups according to the 2017 WHO histological typing. Data which consisted of age, sex and site were analyzed using SPSS for Window (version 20.0; SPSS Inc., Chicago, IL) and frequency tables were computed.

**Results:**

A total of 582 OTs were recorded and reviewed, benign OTs were 573 (98.5%) cases and malignant OTs were 9 (1.5%) cases. Of the benign OTs, the epithelial OTs were the commonest (500; 86%) while the benign mixed OTs were the least frequent (21; 3.6%). The mean age was 30±14 years (age range of 3–77years) and the peak age was in the third decade (197; 33.8%) of life. There was slight male gender and strong mandibular site predilection. Ameloblastoma, was the most frequent OT and it accounted for 75.5% of the OTs, followed by adenomatoid odontogenic tumour (8.1%) and odontogenic myxoma (7.2%). Malignant OTs accounted for 1.5% of the OTs.

**Conclusions:**

OTs show a geographic variation with tendency for prevalence of the epithelial OTs in Africa. Ameloblastoma has a high prevalence among Nigerians and is the most common OTs in Africa. Prevalence of odontoma is relatively low in developing African countries like Nigeria when compared to the prevalence in developed countries.

** Key words:**Odontogenic tumours, WHO classification, Nigerians.

## Introduction

Odontogenic tumors (OTs) constitute a heterogeneous group of diseases arising from the tooth-forming tissues or their remnants (1). They possess both diverse clinical and histopathologic features and may be derived from epithelial, mesenchymal (ectomesenchymal) elements or could be mixed.

The first internationally accepted classification system for OTs was published in 1971 (2) by the World Health Organization (WHO). This was reviewed in 1992 (3), updated in 2005 (4) and the fourth edition which is the most recent WHO classification of OTs was published in January 2017 (5). This newly updated classification adopts a simpler format of the germ cell layer of origin such as epithelial, mesenchymal (ectomesenchymal) and mixed odontogenic tumors. And just like the earlier editions, mainly divided odontogenic tumors into two categories, based on biologic behavior as malignant and benign. It however updates and restores odontogenic cysts that were previously eliminated from the 2005 edition. Keratocystic odontogenic tumour (KCOT) and calcifying cystic odontogenic tumour (CCOT) were ‘renamed/reclassified’ as odontogenic keratocysts and calcifying odontogenic cysts respectively. The complex and detailed malignant odontogenic tumor classification of the 2005 edition was also made simpler by this 2017 classification. Furthermore, addition of new lesions; primordial odontogenic tumor (POT), cementoossifying fibroma (COF) and sclerosing odontogenic carcinoma (SOC) were also added in the recent classification (5). POT is a rare lesion with about ten cases reported in the literature. It is a benign mixed odontogenic tumour seen mostly in early adolescent with preferential mandibular site. COF of the jaws was previously regarded as a composite of ossifying fibroma, classified as neoplastic type of fibroosseous lesions. It is now re-designated as a benign mesenchymal OTs. SOC is a new entity under the classification of malignant OT with only about 10 cases reported so far in the scientific literature.

Several world-wide reports on OTs were based on the 1992 WHO histological classifications (6). Reports from African (7-17), Asian (6,18-27), European (28-31), North American (32-35) and South American (36-39) studies show regional differences in the relative frequencies of OTs among different population samples. This may be attributed to genetic and cultural diversity in the different geographical locations. Previous Nigerian studies (8,9,12) that were based on 1992 WHO classification recorded ameloblastoma (59-74%), odontogenic myxoma (6-16%) and adenomatoid odontogenic tumour (2-13%) as the three most prevalent OTs.

Although many studies (7-9,12,14) have been conducted on OTs in Nigerian, all but one (7) of these was based on the 1992 WHO classification and all were single-center studies. The present study review 582 cases of odontogenic tumors histologically diagnosed in four Nigerian tertiary hospitals and the OTs were based on the 2017 WHO histological typing. The prevalence of these tumours among Nigerians was analyzed and the results compare with reports from world-wide studies.

## Material and Methods

A 12-year (2004-2015) retrospective review of OTs was carried out. Information from case files and histopathology records of patients with OTs were retrieved from 4 major tertiary hospitals in Nigeria (the hospitals in Ile-Ife and Lagos are located in Southern Nigeria, serving 7 states with the population of about 27,820,934 while the hospitals in Kano and Sokoto are located in Northern Nigeria and serving 8 states with the population of about 36,777,369). Information retrieved consisted of demographic data on age, sex, site, radiological presentation and definitive histological diagnosis. OTs were further re-classified according to the 2017 WHO histological typing. The benign OTs were categorized into three groups; group I (Odontogenic epithelium), group II (Odontogenic mesenchyme) and group III (Mixed odontogenic tumors) while the malignant OTs were categorized into Ameloblastic carcinoma, Primary intraosseous carcinoma and odontogenic sarcoma. Data was analyzed using SPSS for Window (version 20.0; SPSS Inc., Chicago, IL) frequency tables were computed.

## Results

A total of 582 OTs were recorded over a 12-year period. Benign OTs accounted for 98.5% (573 cases) while malignant OTs (9 cases) accounted for 1.5% in this series. Of the benign OTs, the epithelial OTs (benign histologic group I) were the most frequently observed (500 cases) and accounted for 86%, while the benign mixed OTs (benign histologic group II) were the least frequently observed (21 cases) and accounted for 3.6%. Ameloblastoma which was the most frequently observed lesion accounted for 75.5% of OTs and 76.6% of benign OTs followed by adenomatoid odontogenic tumour (8.1% of OTs and 8.2% of benign OTs)  Table 1. Malignant OTs accounted for 1.5% of OTs ( Table 1). Mean age of patients with OTs was 30.16 (±13.60) years (age range of 3–77years). OTs were most frequently observed among patients in the 3rd decade of life (197 cases; 33.8%) and least among elderly patients (6 cases; 1.0%) with ages within the 8th decade and above. There was a slight male gender and strong mandibular site predilection for the OTs (M:F gender ratio and mandible-maxilla site ratio of 1.2:1 and 4.9:1 respectively)  Table 2. Unlike the other benign OTs, Adenomatoid odontogenic tumour showed maxillary site predilection (70.2%). Seventy-six OTs were recorded in children (aged 15 years and below), accounting for 13.1% of the total OTs. All were benign, most (54;71.1%) were in the second decade of life, ameloblastoma (40;52.6%) constituted more than half of the OTs seen in the paediatric population. The relative percentages of OTs in selected studies across Africa, Asia, South America, North America and Europe is shown in  Table 3.

**Table 1:**
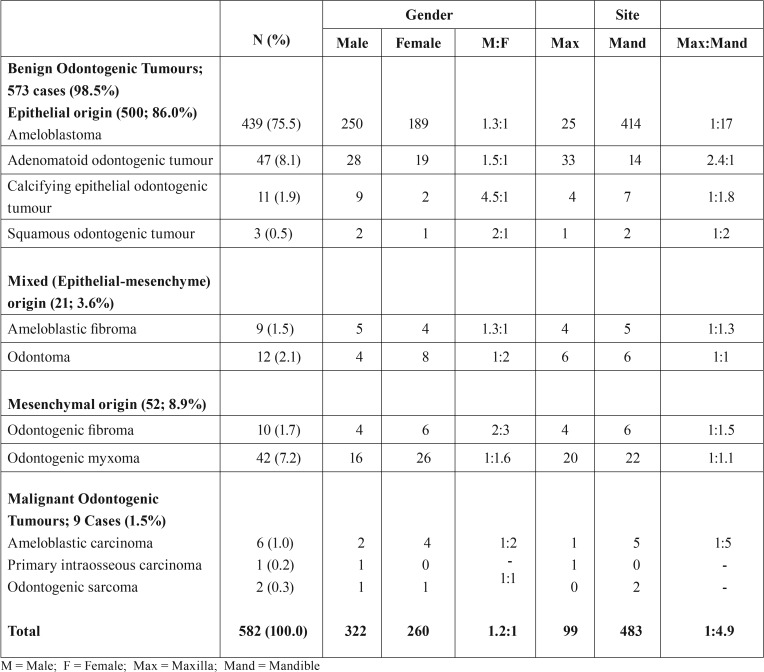
Frequency, gender and site distribution of odontogenic tumours by diagnostic type.

**Table 2:**
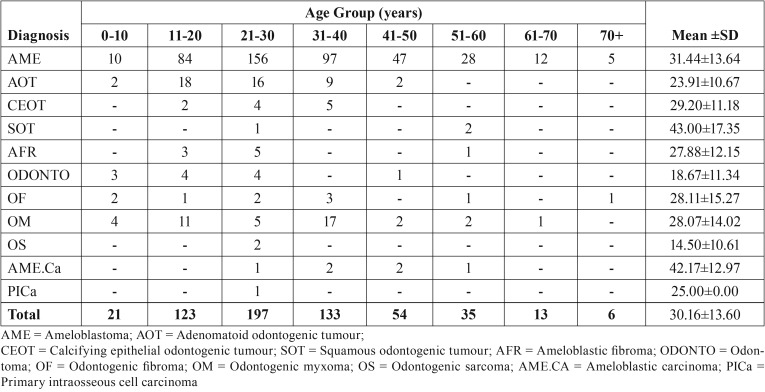
Age distribution of patients with odontogenic tumours.

**Table 3:**
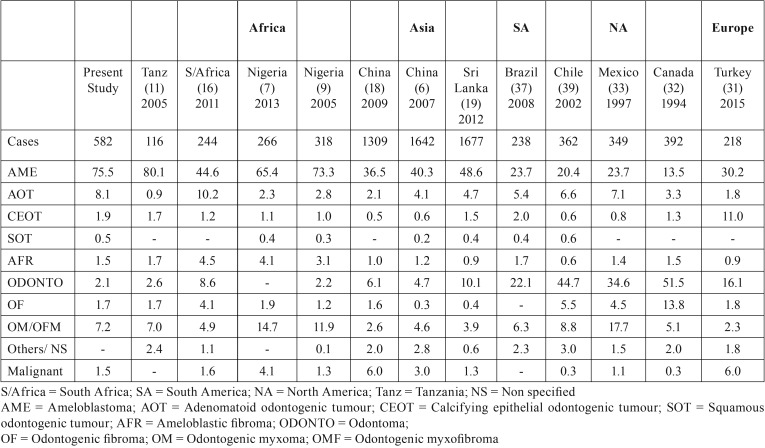
Relative percentage of odontogenic tumours in selected report and the present study.

## Discussion

Several studies (7-9,12,14) have been conducted in Nigeria on OTs. A large number of these studies were single-center studies and therefore may not provide the accurate prevalence of OTs in Nigeria. Our series is a multi-centered study that compiled data from 4 major tertiary referral hospitals in Nigeria. Observation from the present series shows a high prevalence of benign OTs and a very low prevalence of malignant OTs in Nigerians. This agrees with reports from previous studies (9,16,19,30,33) though the observed frequency of occurrence of 1.5% for malignant OTs in this study is lower than reports of 3.0% (Western China) (6), 4.1% (Ibadan, Nigeria) (7), 3.4% (Lagos, Nigeria) (8), 1.6% (South Africa) (16) and 6.0% (Northern China) (18).

Ameloblastoma (75.5%) was the most common type of OT observed followed by AOT (8.1%) and odontogenic myxoma (7.2%). Nigerian studies (8,9,12) based on 1992 WHO classification recorded ameloblastoma (59-73.3%), odontogenic myxoma (6.5-16%) and AOT (2.8-13%) as the three most common OTs whereas a recent Nigerian study (7) based on 2005 WHO classification reported ameloblastoma (65.4%) and odontogenic myxoma (14.7%) as the two most common OTs. The report of high frequency of occurrence for ameloblastoma and low frequency of occurrence for odontoma are consistent with reports from previous African (8,9,11,16) and Asian (6,21) studies. Reports of high prevalence for odontoma from European (30,31) South American (33,36,37,39) and North American (32) studies have been documented with the highest prevalence in North America (51.5%) (32). We report a low frequency of odontoma in Nigerians, a pattern of occurrence similar to reports from previous African (8,9,11,16) and Asian (6,21) studies. This low frequency of odontomas observed may possibly be attributed to underreporting by patients and dentists with consequent dearth of records of odontoma cases. The positively benign self-limiting nature of the growth of odontoma may result in patients not seeking treatment or non-documentation of cases by general dentists.

The observed slight male gender preponderance for OTs though similar to reports by Lawal *et al.* (7) and Adebayo *et al.* (9) but contrasts with female gender preponderance reports from Brazil (36), Chile (39) and Mexico (33). OTs showed a peak incidence in the third decade of life, which was probably related to the marked prevalence of ameloblastoma in this age range. The strong mandibular site predilection presently observed agrees with reports from previous Nigerian studies (7,8,9,12,14) which show mandible: maxilla site ratios to range from between 2.9:1 and 5.7:1. This however differs from equal site predilection reports from South American (33,36,39) and European (30) studies which may be as a result of the different prevalence of ameloblastoma in these areas. Ameloblastoma occurred in all age groups and presented with a peak incidence in the third decade. The mean age of 30.16±13.60 years is similar to age reports from other studies (6,7,9,10,12,36,39) that report mean age ranging between 27.73 to 37.41 years. Observed male gender preponderance for ameloblastoma agrees with reports from previous Nigerian studies (7,9,12) although a slight predilection for females was documented from Turkish (30) and South American studies (33,39). The strong mandibular site predilection for ameloblastoma is consistent with reports from studies in the scientific literature (6-9,12,14,29,30). AOT was the second most prevalent OTs in our series and it showed a site predilection for the maxilla, this is consistent with documented reports from previous studies (7,11,33) however, other studies (6,18,30,39) have reported an equal mandibular-maxillary site distribution. The male gender predilection observed for AOT agrees with reports from previous Nigerian (9) and Chilean (39) studies but differ from other reports (6,7,8,14,33,36).

Odontogenic myxoma (OM) is a relatively rare, locally invasive benign tumour of mesenchymal origin. It is reported to account for between 4.7–17.7% of OTs (8,9,11,12,14,30,32,33,36,39) ( Table 3). This study recorded a prevalence of 7.2% which agrees with previous Nigerian (6.5%) (8) and Brazilian (6.3%) (37) studies. There were 3 odontogenic fibromas and 15 odontogenic myxomas seen in those within the first two decades of life in our series which has similarly been documented as the most common age group for primordial odontogenic tumour. POTs however are composed of variably cellular loose fibrous tissue with areas resembling the dental papilla, entirely surrounded by cuboidal to columnar epithelium and resembling the internal epithelium of the enamel organ 40. The prevalence of OTs in Nigerian children appears to resemble the adult pattern, the epithelial OTs were the commonest (55; 72.4%) while the mixed OTs were the least frequent (9; 11.8%) and ameloblastoma (52.6%) was the most frequently observed lesion, followed by adenomatoid odontogenic tumour (17.1%) and odontogenic myxoma (13.1%), this is similar to previous Nigerian reports (8,12).

Our series further confirms that malignant OTs are rare especially among Nigerians. Perusal of the scientific literature shows that previous African studies (9,16) report a similar low frequency of occurrence which range from between 1.3%-1.6% . This is however slightly higher than reports of 0.3%-1.1% from American (32,32,36,39) and European studies (30) but much lower than reports from Asian studies (6,18) that document frequencies as high as 3.0-6.0%.

Conclusions

OTs show a geographic variation with tendency for prevalence of the epithelial OTs in Africa. There was a slight male gender and strong mandibular site predilection for OTs among Nigerians. Ameloblastoma is the most common OTs in Nigerians and odontomas are rare among Africans but have been reported to occur more in Europe and America. Malignant tumours derived from odontogenic tissues rarely occur in Nigerians.
